# Body Size, Growth and Life Span: Implications for the Polewards Range Shift of *Octopus tetricus* in South-Eastern Australia

**DOI:** 10.1371/journal.pone.0103480

**Published:** 2014-08-04

**Authors:** Jorge E. Ramos, Gretta T. Pecl, Natalie A. Moltschaniwskyj, Jan M. Strugnell, Rafael I. León, Jayson M. Semmens

**Affiliations:** 1 Institute for Marine and Antarctic Studies, University of Tasmania, Hobart, Tasmania, Australia; 2 School of Environmental and Life Sciences, University of Newcastle, Ourimbah, New South Wales, Australia; 3 Department of Genetics, La Trobe Institute for Molecular Science, La Trobe University, Bundoora, Victoria, Australia; University of Sydney, Australia

## Abstract

Understanding the response of any species to climate change can be challenging. However, in short-lived species the faster turnover of generations may facilitate the examination of responses associated with longer-term environmental change. *Octopus tetricus*, a commercially important species, has undergone a recent polewards range shift in the coastal waters of south-eastern Australia, thought to be associated with the southerly extension of the warm East Australian Current. At the cooler temperatures of a polewards distribution limit, growth of a species could be slower, potentially leading to a bigger body size and resulting in a slower population turnover, affecting population viability at the extreme of the distribution. Growth rates, body size, and life span of *O. tetricus* were examined at the leading edge of a polewards range shift in Tasmanian waters (40°S and 147°E) throughout 2011. *Octopus tetricus* had a relatively small body size and short lifespan of approximately 11 months that, despite cooler temperatures, would allow a high rate of population turnover and may facilitate the population increase necessary for successful establishment in the new extended area of the range. Temperature, food availability and gender appear to influence growth rate. Individuals that hatched during cooler and more productive conditions, but grew during warming conditions, exhibited faster growth rates and reached smaller body sizes than individuals that hatched into warmer waters but grew during cooling conditions. This study suggests that fast growth, small body size and associated rapid population turnover may facilitate the range shift of *O. tetricus* into Tasmanian waters.

## Introduction

The distribution and abundance of marine species depends on their functional traits and associated biotic factors, i.e. population genetic structure and gene flow [1], physiological limits [2]–[4], phenotypic plasticity [5], dispersal ability [6], [7], and intra and inter-specific interactions [8], [9]. These functional traits and biotic factors are in turn modulated by abiotic factors such as temperature, oxygen and pH [2], [10]. Temperature is by far the easiest abiotic factor to record and therefore the most studied environmental variable. Moreover, all aspects of ectotherm behaviour and physiology are sensitive to environmental temperature [11], and species changes in distribution in response to climate change are thought to be largely driven by fluctuations in temperature [12]. However, there are substantial inter-specific differences in the magnitude of responses to such temperature variability [4], and we have little knowledge about the processes responsible for the vast variation in species responses. Some studies have suggested that in response to ocean warming, marine species with short lifespans, high genetic diversity, high dispersal capacity, e.g. with a planktonic larval stage or high migration potential, and that live near their upper thermal limit may be more able to change their distribution as they track their optimum thermal conditions [4], [11],[13],[14].

Long-term data sets appropriate to examine the response in life history parameters of long-lived species that may be undergoing climate-driven range shifts are rarely available [15]. In contrast, ecologically and commercially important cephalopods [16] may facilitate the examination of such life history parameters as a function of their generally short lifespan [17]. Life histories of cephalopods are extremely flexible under changing environmental conditions [18], largely due to the effect of temperature on growth [19], size at maturity [20], hatchling size [21], as well as social and behavioural aspects of courtship, mating, and egg-laying [22]. The combination of temperature-driven flexibility in life-history and the short lifespan of cephalopods may be critical for their capacity to thrive under ocean warming.

**Figure 1:**
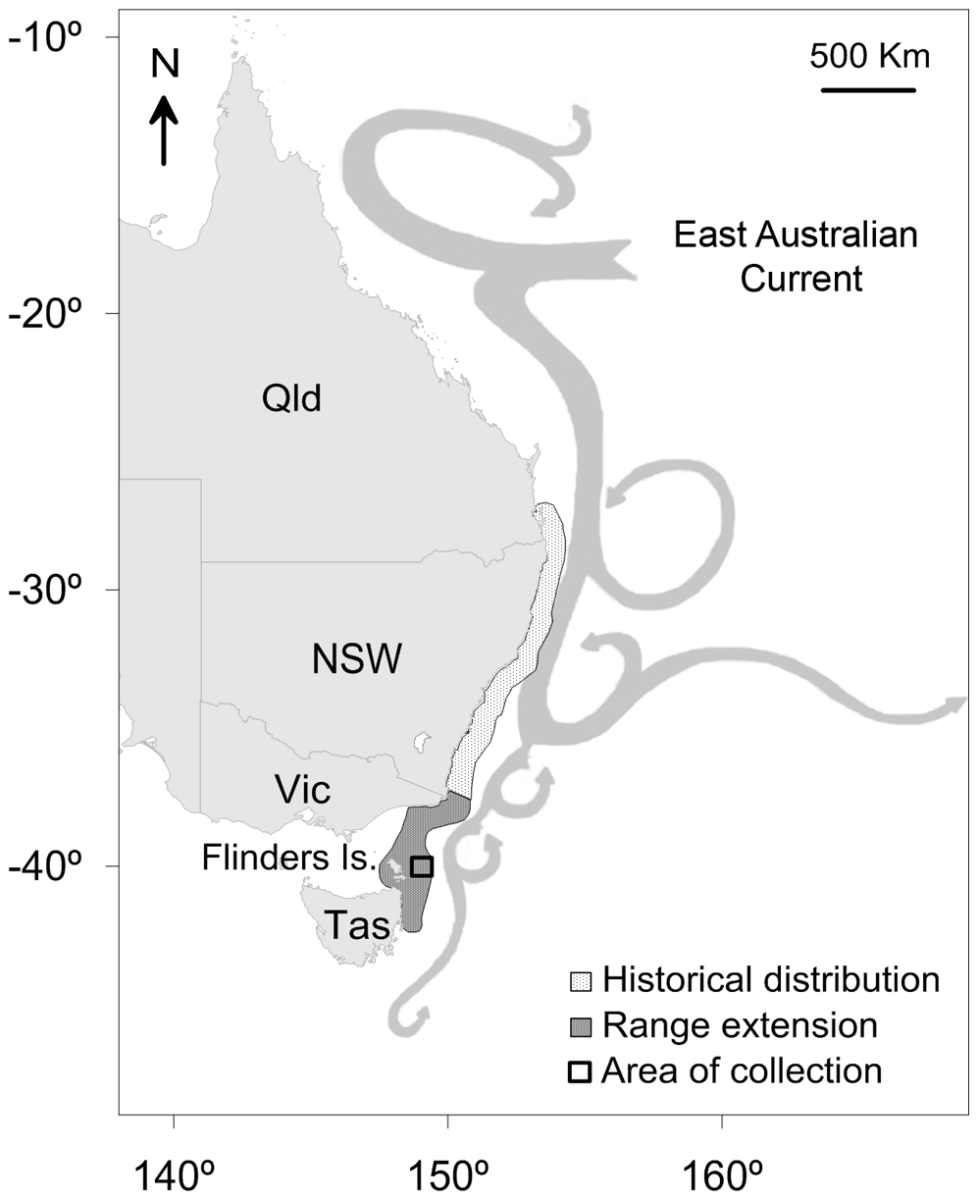
Distribution of *Octopus tetricus* along the east coast of Australia. Collection site off eastern Flinders Island at north-eastern Tasmania, Australia during 2011; Qld  =  Queensland; NSW  =  New South Wales; Vic  =  Victoria; Tas  =  Tasmania.

The gloomy or common Sydney octopus, *Octopus tetricus*, is a merobenthic species with a planktonic paralarval stage of 2.2±0.01 SE mm at hatching size (Ramos et al. unpublished data) that is subjected to ocean currents. The duration of the paralarval stage before settlement is unknown for *O. tetricus* but it is assumed to be similar to that of closely related species [23], i.e. 35–60 days for *O. vulgaris* reared under laboratory conditions [24], [25]. *Octopus tetricus* reaches an approximate arm-span of 2 m [26], [27]. This species is commonly distributed in temperate waters of the east coast of mainland Australia, from southern Queensland to southern New South Wales as suggested by scientific surveys [27], [28]. However, its distribution has extended polewards to south-eastern Australia, along the coasts of Victoria (see [29]) after 2000 approximately, and eastern Tasmania in 2006 (as reported by fisheries data [30] and supported by citizen science monitoring using scientist-verified and geo-referenced photographs [31], [32]; Fig. 1). This polewards shift in distribution, like many others in the same area (e.g. see [32], [33]), is thought to be related to the southern extension of the warm East Australian Current (EAC) [34], [35] and is consistent with expected changes in distribution promoted by climate driven warming [12]. The EAC flows from the southern Coral Sea and reaches the south-east coast of mainland Australia [36]. Over the past 60 years the EAC has extended approximately 350 km further south, along the relatively cool east coast of Tasmania [34], [35]. This extension of the EAC has resulted in the southern Tasman Sea warming at a rate of three to four times the global average, with the ocean temperatures in the region projected to increase by 3°C by 2070 [37].

Like other cephalopods that are key components of trophic webs [16], *O. tetricus* may play an important ecological role in transition of the energy flux from low to high trophic levels. For example, by competing with other octopus species for ecologically and commercially important prey species [38]–[40]. Furthermore, *O. tetricus* is an important target of the octopus fishery in its historical distribution [26] and now also in the leading edge of its range extension, contributing 14% of the octopus catch. Thus, *O. tetricus* provides a good opportunity to examine the influence of environmental variability on life history characteristics, in particular growth, body size and lifespan; and how such characteristics may influence its capacity to become established in the new sections of its range, i.e. if growth rate and population turnover are fast, the establishment of the population will be favoured in the range extension.

Therefore, this study aims to examine the effect of environmental variables on the life history characteristics of *O. tetricus* at the southern edge of the recent range shift, in particular the size structure, growth rates and lifespan, and discuss how these characteristics may potentially influence the current and future establishment of this species in Tasmanian waters.

## Materials and Methods

### Ethics statement

This research was conducted under the University of Tasmania Animal Ethics Committee, permit approval no. A11591. No specific collection permits were required given that *Octopus tetricus* is not an endangered or protected species, and specimens were provided by commercial fishers.

### Collection of wild caught specimens

Octopuses were collected by fishers using black plastic shelter pots, 0.3-m long ×0.1-m high ×0.1-m wide, laid on the seafloor at a depth of 35–46 m off the east coast of Flinders Island, north-eastern Tasmania (approximately 40°S and 147°E; Fig. 1) during January (n = 47), February (n = 78), April (n = 93), May (n = 92), July (n = 45), September (n = 76) and December (n = 96) 2011 on board of the commercial *FV Farquharson*.

The whole animals were frozen on board at −20°C. Specimens collected during February and May 2011 were preserved on board in 80% ethanol. Individuals were dissected in the laboratory and eviscerated total wet weight (TW) and mantle weight (MW) (g) were recorded. Mantle weight was preferred over TW because missing and incomplete arms from many individuals provided an underestimate of TW. However, TW was recorded to facilitate comparison with previous studies. Measurements and weights were recorded to the nearest 0.1 cm and 0.01 g respectively.

To correct weights of −20°C frozen and 80% ethanol preserved individuals, sections of mantle tissue of 1 cm width ×2 cm length of different wild caught individuals were weighed fresh and frozen at −20°C (n = 100) or preserved in 80% ethanol (n = 86). Weight was recorded again after the same period of time that passed between collection of wild caught specimens and weighing in the lab.

Sex and maturity (immature, mature and spent) were determined based on the macroscopic characteristics of the gonads. The maturity scale was modified from previous studies [41], [42].

### Age estimation

**Figure 2:**
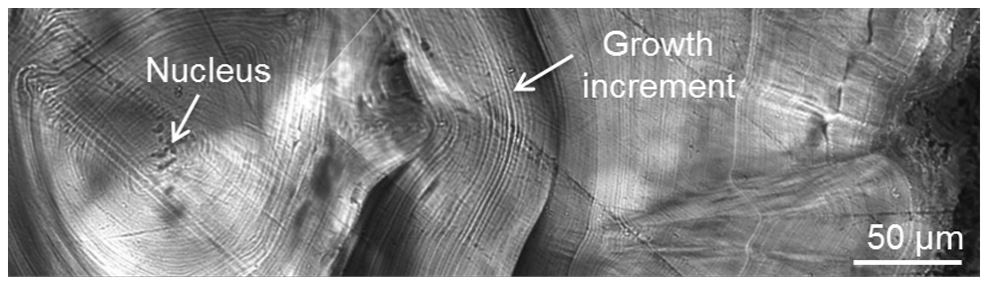
Stylet of *Octopus tetricus*. Microstructure of a stylet's transverse section where growth increments are observed.

Stylets, the vestigial shells of octopods, have recently been used with success to estimate age and growth rates [43]–[48]. Stylets were removed from the mantle of fresh, frozen or 80% ethanol preserved specimens and stored in 70% ethanol. Stylets of all specimens (n = 527) were cut, embedded, ground, and polished following [46] with slight modifications: Two to five pictures, depending on section diameter, were sequentially taken from the nucleus to the edge of the section at either ×100, ×200 or ×400 magnification (Fig. 2) using the software Leica Application Suite (LAS) v. 3.6.0 (Leica Microsystems, Switzerland) with a transmitted-light microscope Leica DM LB2 connected to a digital camera Leica DFC420. Pictures were sequentially stitched together and daily increments identified following [43]. Two non-consecutive increment counts were made by one reader using key counter software (KeyCounter v. 1.1.0) and a third count was carried out by a second reader. Recorded number of growth increments was considered as the mean of the three counts. Stylet sections were discarded (n = 313) if growth increments were not clear along the section, if more than 10% of the section was unable to be counted, and if the three counts differed by more than 10%. The daily periodicity of growth increments was assumed in this study as it has been validated or assumed for holobenthic [43] and merobenthic octopods [46]–[48].

### Oceanographic data

Monthly average Sea Surface Temperature (SST) and Chlorophyll-*a* (Chl-*a*) concentration of the sampling area was obtained from the MODISA satellite imagery at a 4 km scale (http://oceandata.sci.gsfc.nasa.gov/MODISA/Mapped/Monthly/4 km/). Sea surface temperature was considered a valid temperature estimate of the habitats occupied by *Octopus tetricus* because this species was collected at shallow depths (35–46 m) where wind driven mixing is high and tidal currents are strong [49]. Chlorophyll-*a* concentration is an estimator of primary productivity often with strong links to the abundance of higher trophic levels [50], [51].

### Data analyses

Paired sample t-tests were used to assess significant differences between fresh and frozen, and fresh and 80% ethanol preserved samples. Model II linear regressions were conducted when necessary to adjust frozen and 80% ethanol weights so they were comparable to fresh weights. All data was Box-Cox transformed when necessary using the “car” package in R v. 3.0.1 [52], [53]. Normality was determined using Shapiro-Wilk's test and homogeneity of variances evaluated by visual inspection of residual plots.

Hatch month of each individual was back-calculated, by subtracting an individuals estimated age (days) from its date of capture. Hatch months were grouped into a ‘warm season’ including December to May (17.6±0.38°C SE and 0.70±0.04 mg m^−3^ SE, n = 12) and a ‘cool season’ of June to November (13.2±0.30°C SE and 0.90±0.07 mg m^−3^ SE, n = 12) (following [54]). Maximum life span was considered a proxy of population turnover.

The Gompertz, exponential, power and linear growth models were generated for mantle weight of females and males pooled by season of hatching. The 3-parameter Gompertz growth model had the smallest Akaike Information Criterion (AIC) and Akaike weight (wAIC) closest to 1 [55], [56] using the package “qpcR” in R v. 3.0.1 [53], [57] and was identified as the model that best fitted the size (MW) at age data (Table 1). The Gompertz growth model was constructed using the non-linear weighted least square method following [58]:
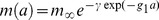



**Table 1:** Akaike Information Criterion (AIC) and Akaike weight (wAIC) for each growth model fitted to mantle weight at age for *Octopus tetricus* that hatched in warm or cool seasons.

	Warm		Cool	
Growth model	AIC	wAIC	AIC	wAIC
Gompertz	−3129.39	1	−13709.12	1
Exponential	−2615.98	<0.0001	−9572.86	0
Power	787.16	0	1229.35	0
Linear	784.14	0	1219.75	0

Individuals were collected at north-eastern Tasmania, Australia during 2011.

Where
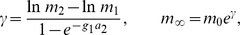


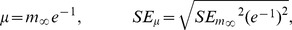



Where *m* is mantle weight (g); *a* is age (days); *m*_∞_ is the asymptote parameter in *m*(*a*) (g); γ is the shape parameter in *m*(*a*); g_1_ is the rate coefficient parameter in *m*(*a*) (day^−1^); *m*_1_ and *m*_2_ are location parameters in *m*(*a*) (g), or predicted mantle weight at minimum or maximum observed age; *a*_2_ is the maximum observed age; *μ* is the inflection point parameter in *m*(*a*) (g) and SE is the standard error. The three parameters to estimate are *m*_∞_, γ and g_1_. The 95% confidence intervals for the coefficients of the Gompertz growth model were estimated by bootstrapping using the package “car” in R v. 3.0.1 [52], [53]. The F-statistics was calculated through an analysis of residual sum of squares (ARSS) to compare Gompertz growth models between genders and among warm and cool hatching seasons [59]. The instantaneous relative rate of growth (*G*) for the Gompertz model was estimated following [58]:
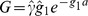



A two-way ANOVA was used to compare differences in MW of mature females, and mature and spent males, as well as to compare differences in age of mature and spent females and mature and spent males among warm and cool seasons of hatching. All statistics and models were carried out using R v. 3.0.1 [53].

## Results

### Body size and life span

**Figure 3:**
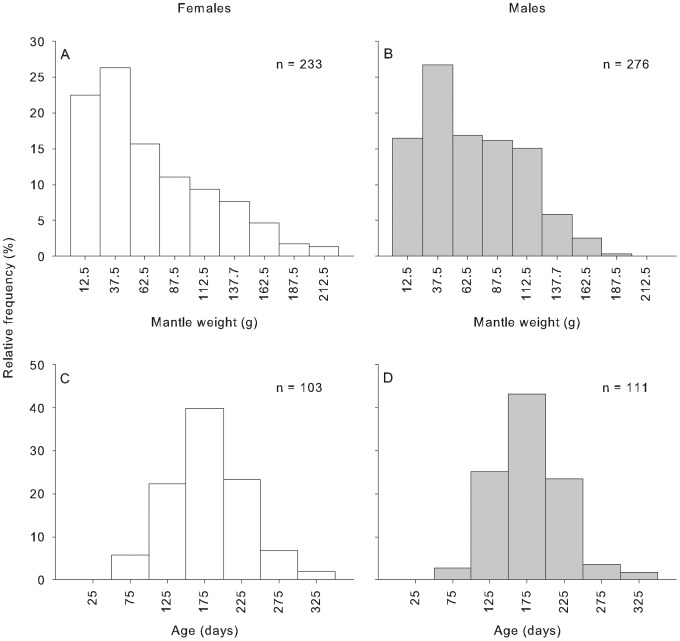
Relative frequency distribution of females and males *Octopus tetricus*. Relative frequency (%) at A–B) mantle weight (g) and C–D) age (days), respectively at the range extension off north-eastern Tasmania, Australia during 2011.

A total of 527 *Octopus tetricus* (250 females and 277 males) were collected during 2011, approximately 40% of which were smaller than 40 g in MW. Mantle weight distribution was not significantly different between females (5–209 g) and males (3–189 g, Fig. 3) (ANOVA, F_(1,505)_ = 0.338, P>0.56); with TW ranging between 60–2260 g for females and 50–2100 g for males. Age of females was not significantly different from age of males (ANOVA, F_(1,212)_ = 0.039, P = 0.84); females were estimated to be 85–308 days (n = 103) of age and males 88–313 days (n = 111, Fig. 3). Maximum life span was 11 months.

### Growth rates, body size and life span at hatch seasons

**Figure 4:**
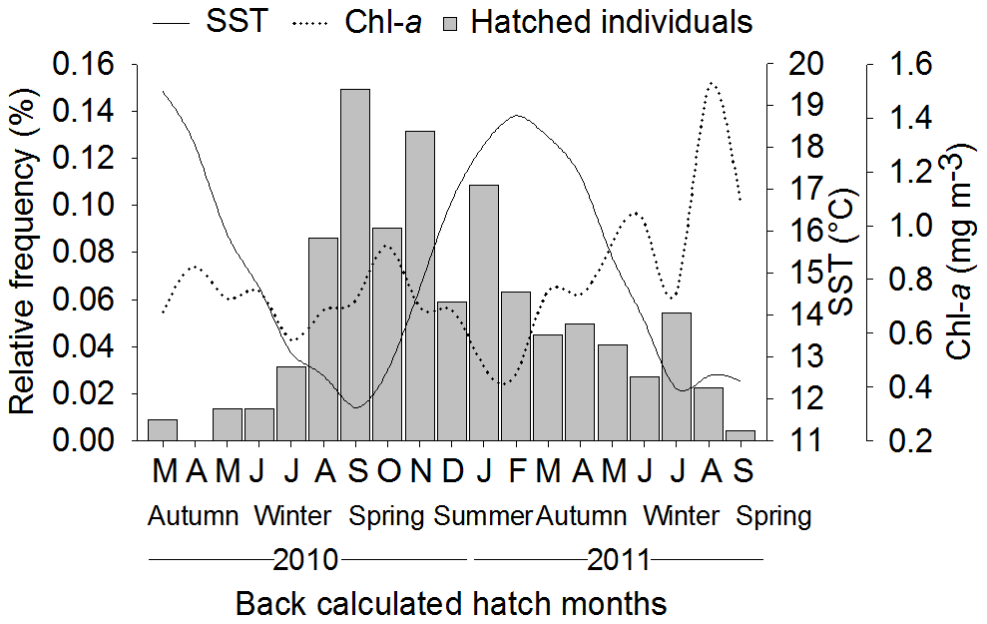
Relative frequency (%) of hatched *Octopus tetricus* (n = 214) from north-eastern Tasmania, Australia during 2011. Warm months are indicated in bold and cool months are indicated in italic.

**Figure 5:**
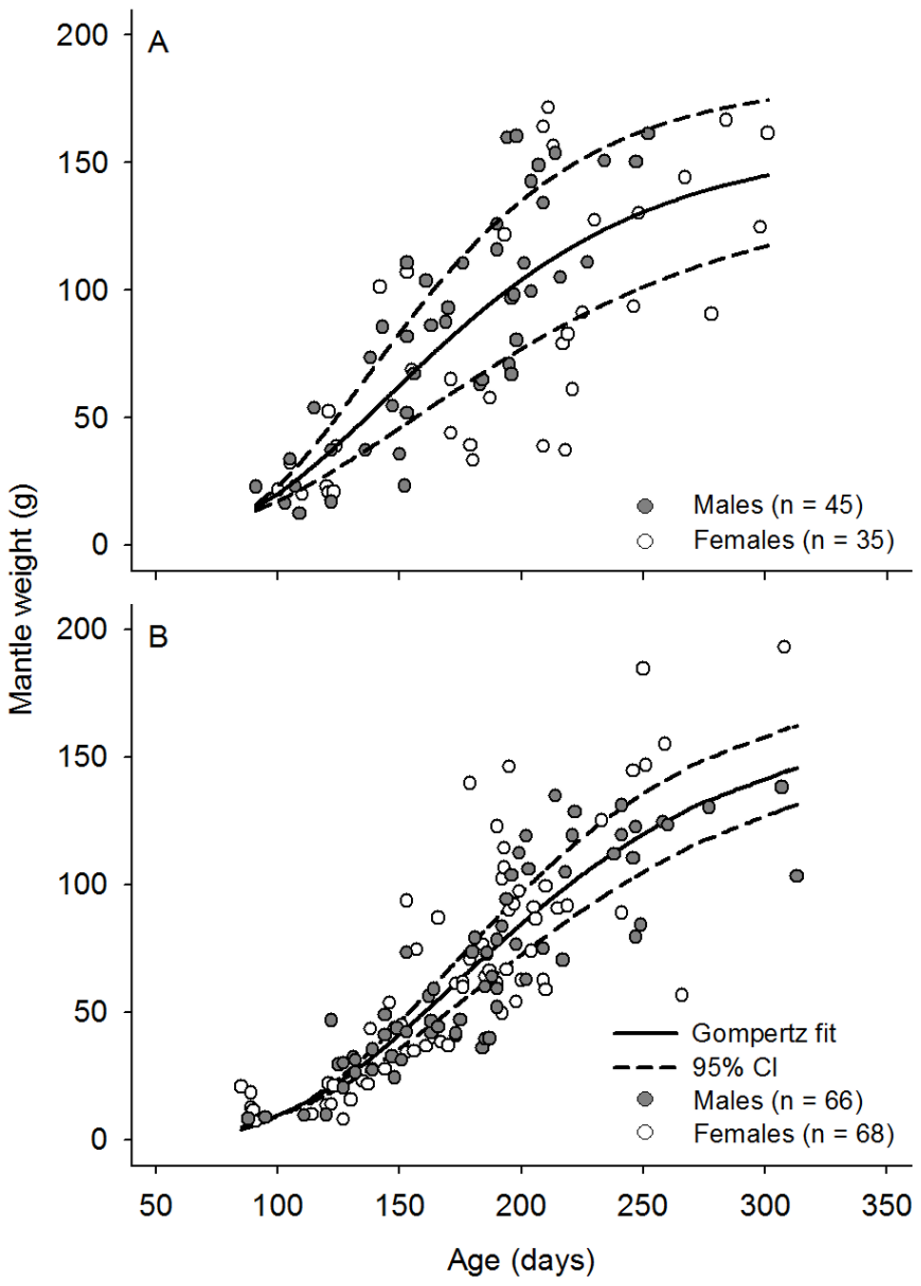
Growth of *Octopus tetricus*. Mantle weight (g) and age (days) data, and fitted 3-parameter Gompertz growth model for *Octopus tetricus* that hatched in A) warm and B) cool seasons at north-eastern Tasmania, Australia. Individuals were collected during 2011 and hatch seasons were back-calculated from growth increments in stylets. CI =  Confidence interval.

Individuals collected in 2011 hatched throughout 2010 and 2011. Greatest numbers of these animals hatched in 2010 when SST was at coolest and Chl-*a* concentration highest (Fig. 4). A second hatching peak was observed in January 2011 when SST was increasing and Chl-*a* concentration was decreasing. The estimated average instantaneous relative growth rate 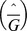 of all *O. tetricus* in north-eastern Tasmania was 0.014±0.0006 SE day^−1^, n = 214 (Table 2). The ARSS indicated that growth models differed between hatching seasons (F_(3,211)_ = 7.03, P<0.0001; Fig. 5). For instance, instantaneous relative growth rate of cool hatched animals was significantly faster than the instantaneous relative growth rate of warm hatched animals (Table 2). Growth models differed only between females and males that hatched in the warm season (F_(3,77)_ = 2.79, P<0.0001).

**Table 2:** Parameter estimates for the 3-parameter Gompertz growth model fitted to mantle weight at age, and instantaneous relative growth rate for backed calculated hatched *Octopus tetricus* from north-eastern Tasmania, Australia collected during 2011.

By hatch season	n	Age (days)	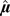 (g)	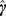	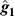 (day^−1^)	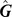 (day^−1^)
w	80	91–301	57.90 (4.93)	10.15 (2.50)	0.016 (0.0023)	0.012 (0.001)
c	134	85–313	61.06 (2.88)	12.20 (1.52)	0.015 (0.0009)	0.016 (0.0009)
By gender						
f	103	85–308	88.77 (13.91)	7.05 (0.76)	0.009 (0.001)	0.013 (0.0006)
m	111	88–313	54.67 (1.09)	13.77 (1.19)	0.017 (0.0007)	0.014 (0.001)
All	214	85–313	63.11 (3.54)	9.81 (1.03)	0.0137 (0.0009)	0.014 (0.0006)

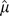
 =  inflection point parameter in mantle weight (age) (g); 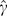 =  shape parameter in mantle weight (age) (g); 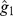 =  rate coefficient parameter in mantle weight (age) (day^−1^); 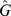 =  instantaneous relative growth rate (day^−1^); c =  cool; w =  warm; f =  females; m =  males. Asymptotic standard errors indicated in parenthesis. Significance for estimated parameters P<0.05.

Approximately 14% of captured females and 44% of captured males were mature or spent. Maximum MW of mature females was significantly different between hatching seasons (ANOVA, F_(1,10)_ = 13.2, P = 0.005), with females that hatched during the warm season heavier (144.89±8.56 SE g, n = 5) than females that hatched in the cool season (94.92±9.83 SE g, n = 7). Similarly, mature and spent males that hatched in the warm season were heavier (120.28±7.24 SE g, n = 19) than males that hatched in the cool season (92.20±7.09 SE g, n = 22; ANOVA, F_(1,39)_ = 7.61, P = 0.009). Considering only mature and spent females, individuals that hatched in the warm season were significantly older (271±11.72 SE days, n = 6) than females that hatched in the cool season (194.75±13.26 SE days, n = 8; ANOVA, F_(1,12)_ = 17.13, P = 0.001). In contrast, age of mature and spent males that hatched in the warm season (196.32±7.15 SE days, n = 19) did not differ significantly from the age of males that hatched in the cool season (218.32±9.49 SE days, n = 22; ANOVA, F_(1,39)_ = 3.26, P = 0.08).

## Discussion

This study demonstrates that *Octopus tetricus* has a fast growth rate, small body size and a short lifespan of approximately 11 months, even at the cooler leading edge of its polewards range extension. These characteristics correspond to an *r*-selected life history strategy, which would facilitate the apparent rapid population expansion of this species and assist the ‘invasion’ into new environments [60], [61]. Fast growth rates and short lifespan, combined with successful reproduction, i.e. mating, high fecundity and production of viable embryos (Ramos et al. unpublished data), may underpin a capacity for *O. tetricus* to quickly increase the size of the emerging population in the zone of the range extension. Additionally, such a short lifespan and associated high population turnover may give *O. tetricus*, most likely an efficient generalist predator at the population level like most other octopus species [62], [63], a competitive advantage in the short term (see [64]) over the longer-lived species already found within the new range area.

If food supply is not limited, octopuses from cooler waters are expected to grow slower during the exponential phase of growth and reach maturity at larger sizes compared to octopuses from warmer waters [65], [66]. In contrast, most individuals collected in the relatively cooler Tasmanian waters during 2011 (annual average 15.3±2.4°C SD, n = 12 months), were quite small (< approx. 400 g TW) with the maximum of 2.3 kg TW measured, compared with larger individuals (>3 kg of TW, S. Montgomery pers comm) reported from the warmer New South Wales waters (annual average 20.3±2°C SD, n = 12 months). Life history characteristics, i.e. growth rates, body size and life span, may differ over the distribution of a species, and may even diverge at the extension of the species distribution [61] possibly a function of reduced genetic diversity [67], or altered as an adjustment to the new physical environment [11], or to different community interactions [68]. For example, body size may be smaller in the region of range extension even though life-history theory predicts body size should be larger in cooler waters. Alternatively, the use of shelter pots may have led to aggregation of mature females or limited the body size of *O. tetricus* collected in Tasmanian waters. In contrast, the use of trawl nets in New South Wales would not lead to aggregation of mature females or limit the body size of captured octopuses. However, additional evidence suggests that the age at sexual maturity (206±26 days SD, n = 214; Ramos et al. unpublished data) and time for egg laying and embryo development in Tasmania (∼60 days; unpublished data) fits within the estimated life span (∼11 months) of *O. tetricus*. So it is likely that a reasonable size range has been measured and maximum body size and life span has not been underestimated in the range extension. This is further supported by the similar lifespan of other merobenthic octopods, e.g. *O. cyanea* (11 months [48]), *O. vulgaris* (12–15 months [69]–[71]), or *O. bimaculoides* (14 months [65]).

A short lifespan can facilitate rapid population turnover. Selection acts on biological traits of every generation [72]; thus, favoured genotypes are likely to be selected more often in species with shorter generation times [73], [74] due to greater probability of occurrence of mutations or formation of new gene complexes [75]. In this sense, it is possible that the combined effects of small body size, short life span, and likely rapid adaptation to environmental changes and biotic pressures may allow exploitation of niches, which may facilitate the establishment of *O. tetricus* in the leading edge of the range shift into Tasmanian waters.

The 3-parameter Gompertz growth model was an appropriate fit for size at age of *O. tetricus*. This model has adequately described non-linear relationships for growth estimations for other cephalopods taxa, e.g. squids [58], [76], and is simpler than other models, i.e. the 4-parameter Schnute growth model [58]. To our knowledge, this is the first study that suggests the Gompertz model as the best fit for growth of an octopod. Therefore, it is not possible to compare with growth models of other octopods. In contrast, the instantaneous growth rate estimated in this study is comparable only to those estimated during the exponential growth of octopods using the equation (*G* =  (lnW_2_–lnW_1_)/(t_2_–t_1_)) by [19], [58]. *Octopus tetricus* shows similar growth rates compared to wild caught octopods in their historical distribution, e.g. 0.011±0.003 SE day^−1^, n = 628 for *O. vulgaris* [69], and octopods in captivity, e.g. 0.014±0.0004 SE day^−1^, n = 18 for *O. pallidus* [77], 0.018±0.002 SE day^−1^ for *O. maya*, n∼40 [78], and 0.036±0.005 SE day^−1^, n = 84 for *O. bimaculoides* [65]. Similarity of growth rates suggests that the growth rate of *O. tetricus* in the area of the range extension may not be negatively impacted, still allowing fast growth rates and promoting a short life span and rapid population turnover. In this sense, fast growth rates may facilitate the establishment of *O. tetricus* at the range extension in Tasmanian waters.

### Influence of environmental factors on growth rates

**Figure 6:**
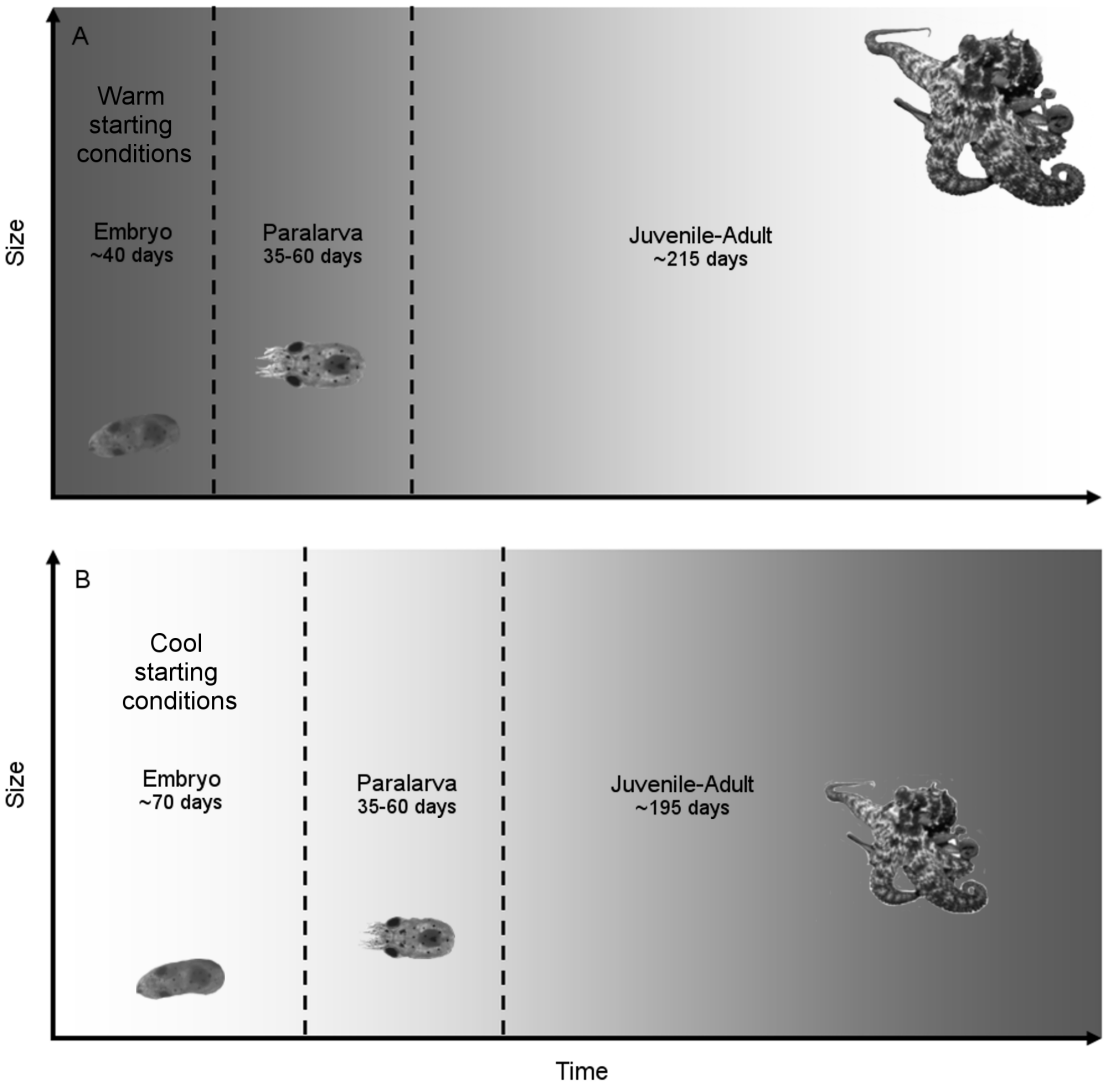
Life cycle of *Octopus tetricus*. A) Octopuses that hatch in warm temperatures have a shorter embryonic phase and likely have faster growth during the exponential phase (embryo and paralarva). Decreasing temperatures during the juvenile and adult phases lead to slower growth resulting in longer life span and larger body size. Note the gradient of temperature from warm (dark grey) to cool (light grey). B) Octopuses that hatch in cool temperatures have a longer embryonic phase and slower growth during the exponential phase (embryo and paralarva). Increasing temperatures during the juvenile and adult phases lead to faster growth resulting in shorter life span and smaller body size. Note the gradient of temperature from cool (light grey) to warm (dark grey). Photo of adult *O. tetricus* by Rick Stuart-Smith.

The estimated frequency of hatched individuals may have been masked by gaps in the collection of specimens during some months, in addition to natural processes such as predation, natural mortality, etc. that were not accounted in this study. With this in mind, our results show that *O. tetricus* hatched throughout the year, with greater number of hatched individuals during the cool and highly productive season. Individuals that hatched in cool and under higher Chl-*a* concentration experienced warming conditions later in their life. Those individuals grew faster and achieved smaller body sizes than individuals that hatched in warm conditions, under low Chl-*a* concentration, and grew during cooling conditions (Fig. 6). Thus, the initial greater peaks of Chl-*a* (and inferred greater availability of food), combined with increasing temperatures after hatching may be related to the faster growth rate of individuals hatched in cool conditions. Similarly, reproductive events of *O. vulgaris* seem to be synchronized with local events of high productivity [79]–[81] that eventually may benefit hatchlings with greater availability of resources [51]. Likewise, squids have experienced faster growth rates in cool waters and this has been attributed to increased productivity or availability of food (*Todarodes angolensis* [82], *Loliolus noctiluca* [83], *Loligo opalescens* [84]).

Size at age variability was observed as aged increased. Individual growth variability has also been noted in other studies [48], [58], [71], [85]. Such variability in response to environmental factors, particularly to temperature, has been extensively studied in squids [19]. Yet, the relationship of growth in octopods and environmental variability is far from understood [86] and other biological factors such as gender, reproduction, genetics and physiological traits must be considered. For instance, differential growth of females and males has been observed during the slower phase of growth with the start of sexual maturity [19], which may explain the different growth rates observed between females and males that hatched in the warm season. This suggests that SST, availability of food, and probably other environmental and biotic factors influenced growth rates at different levels. Therefore, growth must be studied considering the interaction of environmental and biotic factors, in order to disentangling their individual effects.

This study has provided biological information on age and growth of *O. tetricus* at the leading edge of a recent and rapid range shift. The synchrony of hatching events with environmental conditions, such as availability of higher food concentration and warmer temperatures, appears to have a large influence on growth rates, body size and life span of this octopus. Instantaneous growth rates of *O. tetricus* were similar to those of other octopods within their historical range of distribution or reared in captivity. The estimated life span of 11 months may allow *O. tetricus* to cope with environmental variability and possibly facilitate exploitation of available niches. Additional studies on population linkages, reproductive biology (e.g. Ramos et al. unpublished data), trophic ecology, thermal physiology, and dispersal or migration capacity are essential to develop a more complete understanding of the capacity of a species to alter its range and comprehend the biological and ecological mechanisms that underpin that extension.

## References

[1] Holt RD (2003) On the evolutionary ecology of species' ranges. Evol Ecol Res 5: 159–178.

[2] Rosa R, Seibel BA (2008) Synergistic effects of climate-related variables suggest future physiological impairment in a top oceanic predator. Proc Natl Acad Sci USA 105: 20776–20780 10.1073/pnas.0806886105 19075232 PMC2634909

[3] Calosi P, Bilton DT, Spicer JI, Votier SC, Atfield A (2010) What determines a species' geographical range? Thermal biology and latitudinal range size relationships in European diving beetles (Coleoptera: Dytiscidae). J Anim Ecol 79: 194–204 10.1111/j.1365-2656.2009.01611.x 19761459

[4] Sunday JM, Bates AE, Dulvy NK (2012) Thermal tolerance and the global redistribution of animals. Nat Clim Change 2: 686–690 10.1038/NCLIMATE1539

[5] Engel K, Tollrian R, Jeschke JM (2011) Integrating biological invasions, climate change and phenotypic plasticity. Commun Integr Biol 4: 247–250 10.4161/cib.4.3.14885 21980551 PMC3187879

[6] Cowen RK, Sponaugle S (2009) Larval dispersal and marine population connectivity. Ann Rev Mar Sci 1: 443–466 10.1146/annurev.marine.010908.163757 21141044

[7] Travis JMJ, Delgado M, Bocedi G, Baguette M, Barton, et al (2013) Dispersal and species' responses to climate change. Oikos 122: 1532–1540 10.1111/j.1600-0706.2013.00399.x

[8] Colautti RI, Grigorovich IA, Grigorovich A, MacIsaac HJ (2006) Propagule pressure: A null model for biological invasions. Biol Invasions 8: 1023–1037 10.1007/s10530-005-3735-y

[9] Blois JL, Zarnetske PL, Fitzpatrick MC, Finnegan S (2013) Climate change and the past, present, and future of biotic interactions. Science 341: 499–504 10.1126/science.1237184 23908227

[10] Calosi P, Turner LM, Hawkins M, Bertolini C, Nightingale G, et al (2013) Multiple physiological responses to multiple environmental challenges: an individual approach. Integr Comp Biol 53: 660–670 10.1093/icb/ict041 23660590

[11] Pörtner HO, Farrell AP (2008) Physiology and Climate Change. Science 322: 690–692 10.1126/science.1163156 18974339

[12] Poloczanska ES, Brown CJ, Sydeman WJ, Kiessling W, Schoeman DS, et al (2013) Global imprint of climate change on marine life. Nat Clim Change 3: 919–925 10.1038/NCLIMATE1958

[13] Parmesan C, Yohe G (2003) A globally coherent fingerprint of climate change impacts across natural systems. Nature 421: 37–42 10.1038/nature01286 12511946

[14] Pinsky ML, Worm B, Fogarty MJ, Sarmiento JL, Levin SA (2013) Marine taxa track local climate velocities. Science 341: 1239–1242 10.1126/science.1239352 24031017

[15] Hoving HJT, Gilly WF, Markaida U, Benoit-Bird K, Brown ZW, et al (2013) Extreme plasticity in life-history strategy allows a migratory predator (jumbo squid) to cope with a changing climate. Glob Change Biol 19: 2089–2103 10.1111/gcb.12198 23505049

[16] Clarke MR (1996) The Role of Cephalopods in the World's Oceans: General Conclusion and the Future. Philos Trans R Soc Lond B Biol Sci 351: 1105–1112.

[17] Mangold K (1983) Reproduction. In: Boyle PR, editor. Cephalopod life cycles, comparative reviews, vol. 2 . Academic Press, London. pp. 157–200.

[18] Pecl GT, Jackson GD (2008) The potential impacts of climate change on inshore squid: biology, ecology and fisheries. Rev Fish Biol Fisheries 18: 373–385 10.1007/s11160-007-9077-3

[19] Forsythe JW, Van Heukelem WF (1987) Growth. In: Boyle PR, editor. Cephalopod life cycles, comparative reviews, vol. 2 . Academic Press, London. pp. 135–156.

[20] Jackson GD (2004) Advances in defining the life histories of myopsid squid. Mar Freshw Res 55: 357–365 10.1071/MF03152

[21] Pecl GT, Steer MA, Hodgson KE (2004) The role of hatchling size in generating the intrinsic size-at-age variability of cephalopods: extending the Forsythe Hypothesis. Mar Freshw Res 55: 387–394 10.1071/MF03153

[22] Hanlon RT, Messenger JB (1996) Cephalopod behaviour. Cambridge University Press, Cambridge.

[23] Guzik MT, Norman MD, Crozier RH (2005) Molecular phylogeny of the benthic shallow-water octopuses (Cephalopoda:Octopodinae). Mol Phylogenet Evol 37: 235–248. 16009571 10.1016/j.ympev.2005.05.009

[24] Villanueva R (1995) Experimental rearing and growth of planktonic *Octopus vulgaris* from hatching to settlement. Can J Fish Aquat Sci 52: 2639–2650.

[25] Carrasco JF, Arronte JC, Rodriguez C (2006) Paralarval rearing of the common octopus, *Octopus vulgaris* (Cuvier). Aquac Res 37: 1601–1605.

[26] Scandol J, Rowling K, Graham K, editors (2008) Octopus (*Octopus* spp.). In: Status of fisheries resources in NSW 2006/2007, NSW Department of Primary Industries, Cronulla. pp. 193–196.

[27] Norman M, Reid A (2000) A guide to Squid, Cuttlefish and Octopuses of Australasia. CSIRO Publishing, Collingwood, VIC.

[28] Edgar GJ (2000) Australian Marine Life: The plants and animals of temperate waters. Reed New Holland Publishers, Sydney, NSW.

[29] Villanueva R, Norman D (2008) Biology of the planktonic stages of benthic octopus. Oceanogr Mar Biol, Annu Rev 46: 105–202.

[30] Tasmania Wild Fisheries Management Branch, Tasmania Department of Primary Industries and Water (2009) Scalefish Fishery Management Plan Review. Octopus fishery. Dept. of Primary Industries and Water, Hobart, Tas.

[31] Range Extension Database and Mapping Project, REDMAP. 2013. Available: http://www.redmap.org.au. Accessed 4 Sept 2013.

[32] Johnson CR, Banks SC, Barrett NS, Cazassus F, Dunstan PK, et al (2011) Climate change cascades: Shifts in oceanography, species' ranges and subtidal marine community dynamics in eastern Tasmania. J Exp Mar Biol Ecol 400: 17–32 10.1016/j.jembe.2011.02.032

[33] Last PR, White WT, Gledhill DC, Hobday AJ, Brown R, Edgar GJ, Pecl G (2011) Long-term shifts in abundance and distribution of a temperate fish fauna: a response to climate change and fishing practices. Global Ecol Biogeogr 20: 58–72 10.1111/j.1466-8238.2010.00575.x

[34] Ridgway KR (2007) Long-term trend and decadal variability of the East Australian Current. Geophys Res Lett 34: L13613 10.1029/2007GL030393

[35] Hill KL, Rintoul SR, Coleman R, Ridgway KR (2008) Wind forced low frequency variability of the East Australia Current. Geophys Res Lett 35: L08602 10.1029/2007gl032912

[36] Ridgway KR, Dunn JR (2003) Mesoscale structure of the mean East Australian Current System and its relationship with topography. Prog Oceanogr 56: 189–222.

[37] Poloczanska ES, Babcock RC, Butler A, Hobday AJ, Hoegh-Guldberg O, et al (2007) Climate change and Australian marine life. Oceanogr Mar Biol, Annu Rev 45: 407–478 10.1201/9781420050943

[38] Wolf BM, White RWG (1997) Movements and habitat use of the queen scallop, *Equichlamys bifrons*, in the D'Entrecasteaux channel and Huon River estuary, Tasmania. J Shellfish Res 16: 533–539.

[39] Okei N (1999) Predation by octopus on released abalone. In: Howell BR, Moksness E, Svasand T, editors. 1st International symposium on Stock enhancement and sea ranching. Norwegian Program Utvikling Stimulering Havbeite, Bergen, Norway. p 468–477.

[40] Harrington JJ, Semmens JM, Gardner C, Frusher SD (2006) Predation of trap-caught southern rock lobsters, *Jasus edwardsii* (Hutton, 1875), in Tasmanian waters by the Maori octopus, *Octopus maorum* (Hutton, 1880): Spatial and temporal trends. Fish Res 77: 10–16 10.1016/j.fishres.2005.09.003

[41] Mangold K (1983) *Octopus vulgaris*. In: Boyle PR, editor. Cephalopod Life Cycles, vol. 1 . Academic Press, London. p 335–364.

[42] Dia MA (1988) Biologie et exploitation du poulpe *Octopus vulgaris* (Cuvier, 1797) des cotes mauritaniennes. PhD dissertation. University of West Brittany, Brest, France.

[43] Doubleday Z, Semmens JM, Pecl G, Jackson G (2006) Assessing the validity of stylets as ageing tools in *Octopus pallidus* . J Exp Mar Biol Ecol 338: 35–42 10.1016/j.jembe.2006.06.027

[44] Leporati SC, Pecl G, Semmens J (2008) Reproductive status of *Octopus pallidus*, and its relationship to age and size. Mar Biol 155: 375–385 10.1007/s00227-008-1033-9

[45] Leporati SC, Semmens JM, Pecl GT (2008) Determining the age and growth of wild octopus using stylet increment analysis. Mar Ecol Prog Ser 367: 213–222 10.3354/meps07558

[46] Barratt IM, Allcock AL (2010) Ageing octopods from stylets: development of a technique for permanent preparations. ICES J Mar Sci 67: 1452–1457 10.1093/icesjms/fsq047

[47] Hermosilla CA, Rocha F, Fiorito G, González AF, Guerra A (2010) Age validation in common octopus *Octopus vulgaris* using stylet increment analysis. ICES J Mar Sci 67: 1458–1463 10.1093/icesjms/fsq054

[48] Herwig JN, Depczynski M, Roberts JD, Semmens JM, Gagliano M, Heyward AJ (2012) Using age-based life history data to investigate the life cycle and vulnerability of *Octopus cyanea* . PLOS ONE 7: e43679 10.1371/journal.pone.0043679 22912898 PMC3422261

[49] Sandery PA, Kämpf J (2007) Transport timescales for identifying seasonal variation in Bass Strait, south-eastern Australia. Estuar Coast Shelf Sci 74: 684–696 10.1016/j.ecss.2007.05.011

[50] Ware DM, Thomson RE (2005) Bottom-up ecosystem trophic dynamics determine fish production in the northeast Pacific. Science 308: 1280–1284 10.1126/science.1109049 15845876

[51] Otero J, Álvarez-Salgado XA, González AF, Miranda A, Groom SB, et al (2008) Bottom-up control of common octopus *Octopus vulgaris* in the Galician upwelling system, northeast Atlantic Ocean. Mar Ecol Prog Ser 362: 181–192 10.3354/meps07437

[52] Fox J, Weisberg S (2011) An {R} Companion to Applied Regression, 2^nd^ edn. Thousand Oaks CA: Sage. Available: http://socserv.socsci.mcmaster.ca/jfox/Books/Companion. Accessed 13 July 2013.

[53] R Core Team (2013) R: A language and environment for statistical computing. R Foundation for Statistical Computing, Vienna, Austria. Available: http://www.R-project.org/. Accessed 18 February 2013.

[54] Moreno A, Pierce GJ, Azevedo M, Pereira J, Santos AMP (2012) The effect of temperature on growth of early life stages of the common squid *Loligo vulgaris* . J Mar Biol Assoc UK 92: 1619–1628 10.1017/s0025315411002141

[55] Akaike H (1974) A new look at the statistical model identification. IEEE Trans Automat Contr 19: 716–723 10.1109/tac.1974.1100705

[56] Wagenmakers EJ, Farrell S (2004) AIC model selection using Akaike weights. Psychon Bull Rev 11: 192–196 10.3758/bf03206482 15117008

[57] Spiess AN (2013) qpcR: Modelling and analysis of real-time PCR data. R package version 1.3–7.1. Available: http://CRAN.R-project.org/package=qpcR. Accessed 4 August 2013.

[58] Arkhipkin AI, Roa-Ureta R (2005) Identification of ontogenetic growth models for squid. Mar Freshw Res 56: 371–386 10.1071/mf04274

[59] Chen Y, Jackson DA, Harvey HH (1992) A comparison of von Bertalanffy and polynomial functions in modelling fish growth data. Can J Fish Aquat Sci 49: 1228–1235.

[60] McMahon RF (2002) Evolutionary and physiological adaptations of aquatic invasive animals: r selection versus resistance. Can J Fish Aquat Sci 59: 1235–1244 10.1139/f02-105

[61] Amundsen PA, Salonen E, Niva T, Gjelland KØ, Praæbel, et al (2012) Invader population speeds up life history during colonization. Biol Invasions 14: 1501–1513 10.1007/s10530-012-0175-3

[62] Anderson RC, Wood JB, Mather JA (2008) *Octopus vulgaris* in the Caribbean is a specializing generalist. Mar Ecol Prog Ser 371: 199–202 10.3354/meps07649

[63] Mather JA, Leite TS, Batista AT (2012) Individual prey choices of octopuses: Are they generalist or specialist? Curr Zool 58: 597–603.

[64] Fulton EA (2011) Interesting times: winners, losers, and system shifts under climate change around Australia. ICES J Mar Sci 68: 1329–1342 10.1093/icesjms/fsr032

[65] Forsythe JW, Hanlon RT (1988) Effect of temperature on laboratory growth, reproduction and life span of *Octopus bimaculoides* . Mar Biol 98: 369–379 10.1007/BF00391113

[66] Forsythe JW (2004) Accounting for the effect of temperature on squid growth in nature: from hypothesis to practice. Mar Freshw Res 55: 331–339 10.1071/MF03146

[67] Arenas M, Ray N, Currat M, Excoffier L (2012) Consequences of range contractions and range shifts on molecular diversity. Mol Biol Evol 29: 207–218 10.1093/molbev/msr187 21778191

[68] Shea K, Chesson P (2002) Community ecology theory as a framework for biological invasions. Trends Ecol Evol 17: 170–176 10.1016/s0169-5347(02)02495-3

[69] Domain F, Jouffre D, Caverivière A (2000) Growth of *Octopus vulgaris* from tagging in Senegalese waters. J Mar Biol Assoc UK 80: 699–705 10.1017/S0025315400002526

[70] Katsanevakis S, Verriopoulos G (2006) Seasonal population dynamics of *Octopus vulgaris* in the eastern Mediterranean. ICES J Mar Sci 63: 151–160 10.1016/j.icesjms.2005.07.004

[71] Canali E, Ponte G, Belcari P, Rocha F, Fiorito G (2011) Evaluating age in *Octopus vulgaris*: estimation, validation and seasonal differences. Mar Ecol Prog Ser 441: 141–149 10.3354/meps09399

[72] Lee CE (2002) Evolutionary genetics of invasive species. Trends Ecol Evol 17: 386–391 10.1016/s0169-5347(02)02554-5

[73] Berteaux D, Reale D, McAdam AG, Boutin S (2004) Keeping pace with fast climate change: can Arctic life count on evolution? Integr Comp Biol 44: 140–151 10.1093/icb/44.2.140 21680494

[74] Hoffmann AA, Willi Y (2008) Detecting genetic responses to environmental change. Nat Rev Genet 9: 421–432 10.1038/nrg2339 18463665

[75] Parmesan C (2006) Ecological and evolutionary responses to recent climate change. Annu Rev Ecol Evol Syst 37: 637–669 10.1146/annurev.ecolsys.37.091305.110100

[76] Schwarz R, Alvarez-Perez JA (2010) Growth model identification of short-finned squid *Illex argentinus* (Cephalopoda: Ommastrephidae) off southern Brazil using statoliths. Fish Res 106: 177–184 10.1016/j.fishres.2010.06.008

[77] Semmens J, Doubleday Z, Hoyle K, Pecl G (2011) A multilevel approach to examining cephalopod growth using *Octopus pallidus* as a model. J Exp Biol 214: 2799–2807 10.1242/jeb.051631 21795579

[78] Rosas C, Tut J, Baeza J, Sánchez A, Sosa V, et al (2008) Effect of type of binder on growth, digestibility, and energetic balance of *Octopus maya* . Aquaculture 275: 291–297 10.1016/j.aquaculture.2008.01.015

[79] Oosthuizen A, Smale MJ (2003) Population biology of *Octopus vulgaris* on the temperate south-eastern coast of South Africa. J Mar Biol Assoc UK 83: 535–541 10.1017/S0025315403007458h

[80] Otero J, Gonzalez AF, Sieiro MP, Guerra A (2007) Reproductive cycle and energy allocation of *Octopus vulgaris* in Galician waters, NE Atlantic. Fish Res 85: 122–129 10.1016/j.fishres.2007.01.007

[81] Lourenço S, Moreno A, Narciso L, González AF, Pereira J (2012) Seasonal trends of the reproductive cycle of *Octopus vulgaris* in two environmentally distinct coastal areas. Fish Res 127: 116–124 10.1016/j.fishres.2012.04.006

[82] Villanueva R (1992) Interannual growth differences in the oceanic squid *Todarodes angolensis* Adam in the northern Benguela upwelling system, based on statolith growth increment analysis. J Exp Mar Biol Ecol 159: 157–177 10.1016/0022-0981(92)90034-8

[83] Jackson GD, Moltschaniwskyj NA (2001) Temporal variation in growth rates and reproductive parameters in the small near-shore tropical squid *Loliolus noctiluca*; is cooler better? Mar Ecol Prog Ser 218: 167–177 10.3354/meps218167

[84] Jackson GD, Domeier ML (2003) The effects of an extraordinary El Niño/La Niña event on the size and growth of the squid *Loligo opalescens* off Southern California. Mar Biol 142: 925–935 10.1007/s00227-002-1005-4

[85] Leporati SC, Semmens JM, Pecl GT (2007) Cephalopod hatchling growth: the effects of initial size and seasonal temperatures. Mar Biol 151: 1375–1383 10.1007/s00227-006-0575-yincrease

[86] Semmens JM, Pecl GT, Villanueva R, Jouffre D, Sobrino I, et al (2004) Understanding octopus growth: patterns, variability and physiology. Mar Freshw Res 55: 367–377 10.1071/MF03155

